# The Breakthrough Generations Study: design of a long-term UK cohort study to investigate breast cancer aetiology

**DOI:** 10.1038/bjc.2011.337

**Published:** 2011-09-06

**Authors:** A J Swerdlow, M E Jones, M J Schoemaker, J Hemming, D Thomas, J Williamson, A Ashworth

**Affiliations:** 1 Section of Epidemiology, Institute of Cancer Research, Sir Richard Doll Building, 15 Cotswold Road, Sutton, Surrey SM2 5NG, UK; 2 Breakthrough Breast Cancer Research Centre, Institute of Cancer Research, Fulham Road, London SW3 6JB, UK

**Keywords:** cohort study, breast cancer, aetiology

## Abstract

**Background:**

The rationale, design, recruitment and follow-up methods are described for the Breakthrough Generations Study, a UK cohort study started in 2003, targeted at investigation of breast cancer aetiology.

**Methods:**

Cohort members have been recruited by a participant referral method intended to assemble economically a large general population cohort from whom detailed questionnaire information and blood samples can be obtained repeatedly over decades, with high completeness of follow-up and inclusion of large numbers of related individuals. ‘First-generation’ recruits were women contacted directly, or who volunteered directly, to join the study. They nominated female friends and family, whom we contacted, and those who joined (‘second generation’) nominated others, reiterated for up to 28 generations.

**Results:**

The method has successfully been used during 2003–2011 to recruit 112 049 motivated participants with a broad geographic and socioeconomic distribution, aged 16–102 years, who have completed detailed questionnaires; 92% of the participants gave blood samples at recruitment. When eligible, 2½ years after recruitment, >98% completed the first follow-up questionnaire. Thirty percent are first-degree relatives of other study members.

**Conclusion:**

The ‘generational’ recruitment method has enabled recruitment of a large cohort who appear to have the commitment to enable long-term continuing data and sample collection, to investigate the effects of changing endogenous and exogenous factors on cancer risk.

Cohort studies have been responsible for establishing almost all of the known causes of cancer (Breslow and Day, 1987). Such studies have tended, however, to be expensive, time-consuming and difficult to carry out well with high completeness of follow-up. As a consequence, practical considerations have constrained the studies that have been conducted.

Breast cancer is by far the most common cancer in women in the United Kingdom and many other Western countries. Although many risk factors have been found over the last 50 years, investigation of its aetiology has been difficult, in part because of the very long period of life over which aetiological factors appear to act, ranging from childhood, perhaps even prenatally, to well after menopause. Difficulty has also arisen from the key role of factors that cannot be ascertained by questionnaire or retrospectively – notably breast density, sex hormone and probably growth-related hormone concentrations – and the complexity of the aetiology of this tumour, involving a multiplicity of genetic susceptibility traits, environmental and behavioural factors, as well as endogenous hormones. We therefore reasoned that to conduct a comprehensive investigation of breast cancer aetiology, a cohort design is needed, in particular to ascertain factors that cannot be ascertained retrospectively, but also to avoid recall bias and imprecision. The study would need to recruit some participants from as young an age as possible, gain blood samples and detailed questionnaire information on exposures, both at recruitment and periodically thereafter, and have a high follow-up rate and the ability and consent to retrieve recorded data and stored biological specimens. Many of these factors depend on gaining a high degree of commitment of the study subjects, such that they are willing to give the time and effort to donate blood samples, complete follow-up questionnaires, allow access to their records and materials, and stay engaged in the study. We therefore wished to develop a recruitment method that would find and involve committed individuals.

## Materials and methods

### Recruitment

The Breakthrough Generations Study is a long-term prospective cohort study focussed on potential aetiological factors for breast cancer in women. It has received appropriate ethics committee approval. The study cohort consists of volunteer women aged 16 years or older at entry. They have been recruited from the general population of the British Isles, and were initially identified from three principal sources. The first source was women on the list of supporters of Breakthrough Breast Cancer, the charity who funded the study. Secondly, as a consequence of publicity, especially at the launch of the study in September 2004, tens of thousands of women contacted the study team to express their interest via our website and telephone lines, or less often by personal contact. Thirdly, women who joined the study were asked if they would nominate female friends and family aged 16 years and older who they thought might also be interested in joining. The probands could discuss the study with the nominees, or not, as they wished, before nominating them.

Women from all these sources were mailed an initial invitation letter and information booklet explaining the study, and a form asking whether they would like to be sent the study pack, without obligation. Those who assented were mailed the study pack, which included a questionnaire, information booklet, consent form and blood pack. If they chose to take part, the women completed and returned to us the consent form and questionnaire. A freephone number was provided to answer any queries. For the blood sample, the majority took their blood pack to their general practice, where a 27-ml blood sample was taken and the cohort member then posted the blood tubes, at ambient temperature, to our laboratory. Some blood samples (<10%) were taken by nurses working for the study, and some samples were taken by others – for instance, phlebotomists at the subject's workplace, nurses or doctors in hospitals, or nurses or doctors otherwise known to the subjects.

For hormone and certain other analyses, it is highly desirable that blood samples be centrifuged, aliquoted and frozen down on the day of receipt. Thus, unlike a study solely collecting questionnaires, which can mail tens of thousands of questionnaires within a few days and then store the returns and process them subsequently over several months, a study collecting plasma needs to receive samples at as constant a rate as possible, avoiding peaks that exceed the laboratory's capacity, or dips that waste laboratory staff time and overhead costs. To achieve this, as we could not control when subjects chose to donate blood and post it, we calibrated the mailing-out rates to generate as constant a flow as possible, based on our experience of the time distribution of response times obtained in the initial stages of the study, allowing for day of the week and season of the year.

When premenopausal women joined the study, they were asked that if possible they should present for venipuncture at a standard point in their menstrual cycle, 7 days before they expected their next period, but if this was not possible, nevertheless to send a blood sample taken when practical. When the subjects returned their blood samples, they also returned a form on which they had recorded several variables relevant to the sample, including the time and date the sample was taken, the time the subject woke on that day, the date of their last menstrual period, and whether they were taking various medicines and supplements.

### Recruitment questionnaire

The study questionnaire asked about demographic variables, and factors known or suspected to affect the risk of breast cancer, including reproductive and menstrual history, exogenous hormone intake, exercise, benign breast disease, alcohol, smoking, some dietary variables, chest exposure to ionising radiation, variables related to the woman's own birth, childhood growth and puberty, height, weight, melatonin-related variables (e.g., shift work, exposure to light at night), occupation, socioeconomic variables, family history of cancer, and chronic diseases. We included questions on childhood and adolescent exposures and behaviours, as well as those in adulthood, and where appropriate (e.g., alcohol consumption, exercise) asked about these variables by age, to allow exposure histories to be built. For the same reason, information on selected exposures is being updated in follow-up questionnaires. The questionnaire also asked the women to self-measure, or ask others to measure for them, certain anthropometric variables – weight, waist circumference, hip circumference and arm span.

Information was sought within the questionnaire, and the participant's informed consent was sought, to enable medical and other relevant records to be located and examined for validation of certain data, and to obtain exposure details that were not possible to obtain from the questionnaires. For instance, information and consent were collected to allow examination of the woman's own birth records, mammograms and treatments. The questionnaire was designed for the subjects’ responses to be read by Optical Character Reading (OCR) software via a scanner (Readsoft, 2011), and, where possible, it asked the respondent to give exact replies to numerical questions, in order to maximise data detail, rather than offering multiple-choice boxes to select between pre-formed ranges. The software provides quality control of the OCR by two methods. First, any characters that the software has uncertainty in reading, or is unable to read, are flagged up to a clerical operative to read and enter the correct character. About 8% of completed fields need clerical intervention. Secondly, for alphabetic and numeric characters, but not for ticks, the software shows the operator all of the responses the subject has made for that character (e.g., all the 7s, all the 8s, etc.), for the operator to inspect and decide whether they are all consistent and have been read correctly. In addition, for certain key variables such as date of birth, the software was programmed to compel the operator to read and check the response for each study subject. We also programmed range and validity checks that show the operator for correction, invalid or unlikely values of characters or combinations of characters (e.g., values for ‘month’ greater than 12).

### Blood sample processing

When blood samples taken as described above were received at the laboratory, the samples were centrifuged and divided into 0.5-ml barcoded ‘straws’ for storage, using a MAPI machine (Cryobiosystem, 2011). This provided approximately 27 straws of plasma and 6 straws of buffy coat per participant, which enable 0.5-ml aliquots to be retrieved for analysis without thawing and refreezing of the remainder of the material. The samples were cooled to −80°C in a freezer and subsequently transferred for long-term storage into liquid-nitrogen tanks, where they are held in the vapour phase at <−180^o^C. The samples for each subject were split and stored in two different sites, 40 miles apart, for security. Each straw is barcoded and recorded on a database that we have constructed so that its location and use can be tracked. DNA is extracted from the buffy coat *ad hoc* as needed, using Qiagen DNA Blood Mini Kits (Qiagen, Valencia, CA, USA); about 30 μg of DNA is obtained per straw, on average. Unnormalised stock DNA samples are then stored at −80^o^C in individual 2D barcoded tubes, and are normalised later when required.

### Follow-up

Follow-up data on site-specific cancer incidence, other major disease occurrence, breast disease and cause-specific mortality are obtained via several sources. First, we receive spontaneous reports from cohort members about the occurrence of breast and other cancers, and from their husbands/partners or other relatives about deaths. Secondly, annual newsletters are sent to the cohort members, and if these are returned as undeliverable, we then ascertain vital status using the methods described below and (if alive) track a current address via the sources below. The newsletter also includes a tear-off sheet for participants to notify us if they have changed name or address. Thirdly, at intervals of about 2½ years since enrolment, participants are sent follow-up questionnaires that ask, *inter alia*, about illness since the last questionnaire and which, effectively, ascertain their vital status. (These questionnaires also ascertain changes in exposure variables since entry into the study and extend the range of questions about exposures (e.g., when new scientific questions arise), and the mailing can be used to obtain repeat blood samples). It is intended that follow-up questionnaires will continue for the next 40 years and longer.

When women are lost to postal follow-up at any of the above points, they are traced via several potential sources, namely (i) e-mail, (ii) telephone, (iii) ‘flagging’ on the National Health Service Central Registers (NHSCRs), virtually complete population registers of England, Wales and Scotland on which cancers, deaths, emigrations and other exits are recorded and can therefore be notified to authorised researchers, (iv) the NHS Strategic Tracing Service (now Personal Demographics Service), an online system derived in part from the NHSCR, that enables address changes and deaths in England and Wales to be looked up and (v) enquiries via the friend or family member who recommended the woman as a potential study subject.

When deaths are ascertained via the above sources, we obtain copies of death certificates to ascertain the cause of death. When cancers are reported we obtain diagnostic confirmation and details from cancer registry data and/or by writing to the appropriate clinicians. We routinely obtain histology information from these sources, gain grade information for a large minority and are now trying to obtain hormone receptor information for incident and recent prevalent cases.

### Family relationships

The original referral forms for nomination of potential new participants asked the relationship of the nominee to the nominator, and the first follow-up questionnaire included a question on whether, to their knowledge, any relatives of the participant had joined the study. If either of these sources indicated a potential relationship, records were cross-matched to determine whether both of the relatives had actually joined the study. This should have given virtually complete family linkage, because each link was potentially reported twice, by each member of the relationship.

## Results

### Recruitment

Using the above recruitment methods, we obtained the names of 372 524 women potentially interested in joining the study, of whom 237 203 (64%) replied positively to the initial invitation letter and were sent the study pack. Of these women, 112 049 (47%) have returned the questionnaire and become study members.

Table 1 shows descriptive characteristics of the cohort: 49% were aged 45–64 at recruitment, 42% younger and 9% older; 46% were in the highest socioeconomic group (based on place of residence; CACI, 2009), but there were appreciable numbers in all socioeconomic groups. Likewise, the largest proportion lived in the South of England (43%), but with substantial numbers from other parts of the United Kingdom. The cohort were somewhat more concentrated in middle age, high socioeconomic status and the South of England compared with the national population.

**Figure 1:**
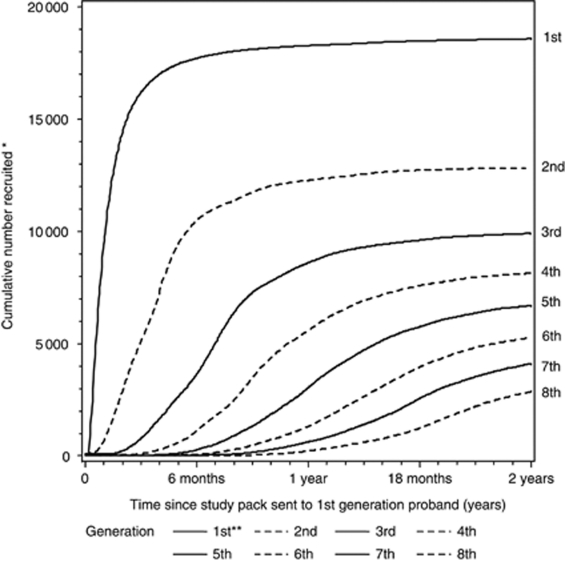
Time course of recruitment of cohort participants, by generation. 1st generation=probands approached directly by the study investigators; 2nd generation=women recommended by the first generation; 3rd generation=women recommended by the second generation, etc. ^*^Recruitment is taken as return of a completed questionnaire. ^**^For practical reasons, in the early part of the study, when publicity provided more recruits than could be processed adequately without delays, a block of first-generation recruits were not asked to nominate further-generation recruits: these women are excluded from the figure.

Figure 1 shows the operation and lag times of the generational recruitment method for the first eight generations of participants. For simplicity of display, the first round of mailing dates are normalised as day 0; in practice they were spread over several calendar years. Each generation lagged the one before it by a mean of 87 days; this lag was slightly shorter at older ages (73 days for those aged 65 and older) than at younger (97 days for those aged under 35), and in later generations (68 days for generations ⩾20) than earlier (91 days in generation 2), but in all instances was in the range 65–100 days.

On average, each woman participating in the study nominated 2.76 candidates to join the study, of whom 0.81 candidates on average, joined. The longest generational chain had reached 28 generations by the time we stopped the generational recruitment process. The nominations for recruitment were skewed, however: 85% of the names were put forward by 30% of the participants, and 48% of the participants suggested no names. The average number of nominations was similar across the generations and by geographical area, but increased with higher socioeconomic status (2.9 for the highest, 2.3 for the lowest) and was greatest for women aged 45–54 years (3.0) and less at younger and older ages than this (e.g., 2.2 for ages 16–24; 1.9 for age 65 and older) (these differences are highly significant, *P*<0.001).

Recruitment ratios (i.e., the ratio of the number of next-generation to the number of previous, ‘proband’, generation participants) increased with age of the proband, from 0.46 for probands aged 16–24 to 0.94 for those aged 55–64, and then decreased slightly (Table 2). The ratio was also generally greater for higher than for lower socioeconomic class probands (0.91 for the highest group, 0.55 for the lowest), but generally showed little variation by geographical area.

The generational recruitment mechanism had the interesting tendency that it produced, after several generations, recruits who were of a fairly similar age and socioeconomic distribution irrespective of the initial distribution of the probands (Tables 3 and 4). Thus, for instance, first-generation probands aged under 35 years produced at the fourth and subsequent generations an intake among whom 18% were aged under 35, 45% aged 35–54 and 37% aged ⩾55, while first-generation probands aged 65 or older produced a ⩾4th generation whose distribution was 14%, 43% and 43%, respectively. This tendency of the recruitment mechanism was less true, however, for geographical area of residence, especially for Scotland: first-generation probands from Scotland provided 55% of ⩾4th-generation recruits from Scotland, whereas first-generation probands from elsewhere gave rise to only 5.5% of recruits from Scotland in the ⩾4th generation.

### Blood samples

Of the women who returned a questionnaire, 102 778 (92%) also gave a blood sample. There are now >3.2 million straws in storage. Including the pilot study, recruitment occurred during 2003–2011, but mainly during 2005–2007, at a rate of about 400–700 per week. The generational recruitment method enabled invitation mailings to be sent out (and names to mail to be available) as a continuous mechanism, promptly as interest was expressed, but with a constant and controlled flow of work through the laboratory. The arrival rate of blood samples never exceeded 760 per week (229 per day). Seventy one percent of samples arrived on the day of venipuncture or the next day, 13% the day after that, 8% after 3 days, 5% after 4 days, and the remaining 4% later (we have found that for female sex hormones, a 2-day lag has only modest effects on concentrations (Jones *et al*, 2007)).

### Family relationships

The generational recruitment method produced a large number of study participants who were related to other participants. In total, 22 977 (21%) were mothers or daughters of other study members, 15 252 (14%) were sisters, including 109 dizygotic and 158 monozygotic twin pairs, and 6991 (6%) were second-degree relatives. There were 15 183 families in the study, with an average of 2.3 study members per family. At recruitment, 13 333 (12%) of participants reported that their mother had had breast cancer, and 4163 (4%) reported at least one sister with breast cancer; the comparable figures for recruits aged 50–64 were 12% and 5%.

### Prevalent breast cancer at study entry

The initial invitation to join the study was open to all women resident in the United Kingdom aged 16 and above, whether or not they had previously had breast cancer. As a consequence, the study includes 6407 women who, the questionnaire showed, had had breast cancer or DCIS before entry into the study. These subjects will for some purposes be analysed separately – for instance, for immediate analysis of the genetics of breast cancer (Fletcher *et al*, 2011), and for cohort analysis of risk factors for second primary, but not first primary, breast cancer. For first primary analyses their reporting of risk factors is potentially biased by their awareness of the prevalent tumour.

### Follow-up

Follow-up questionnaires are being sent to the cohort on a rolling basis: each individual is contacted at fixed periods (2½, 5, 7½, etc., years) related to her original recruitment date. This produces, as for the recruitment, an even workload over time for mailings, especially for laboratory work to process follow-up blood samples without delay. Not all recruits have yet reached their first (2½-year) follow-up date, and the figures below are therefore based on the first 90 000 recruits who have passed this point, plus the further 12 months that has proved necessary to reach a maximum response rate through tracing and reminders. Three hundred and thirty-nine (0.4%) of these subjects have died. Of the 89 661 remaining, 88 596 (98.8%) have contributed a follow-up questionnaire, 315 (0.4%) have emigrated without follow-up questionnaire and 750 (0.8%) others have not yet returned their questionnaire. The follow-up questionnaire percentage among those who have not died is similar by generation, greater for high (99.1%) than for low (97.7%) socioeconomic groups, and somewhat greater at ages 45–64 (99.4%) than at younger (98.4%) or older (98.0%) ages. Follow-up information on vital status has been obtained for 99.5% of the cohort.

## Discussion

Most large cohorts designed to investigate cancer aetiology have been recruited by the investigators contacting potential subjects directly – for instance, specific occupational groups (e.g., Doll and Hill, 1954; Bernstein *et al*, 2002; Nurses Health Study, 2011), members of the general population (e.g., van den Brandt *et al*, 1990; Riboli and Kaaks, 1997; Kolonel *et al*, 2000; UK Biobank, 2011), or health-care groups or trial participants (Buring and Hennekens, 1992; The ATBC Cancer Prevention Study Group, 1994; The Million Women Study Collaborative Group, 1999; Prorok *et al*, 2000; Hays *et al*, 2003). The American Cancer Society have recruited three cohorts by asking volunteers each to recruit a number of their acquaintances (Thun *et al*, 2000). To our knowledge, however, there have not been any large cohorts recruited by multiple ‘generations’ of participant referrals.

We had hoped, and the evidence so far supports this, that recruiting volunteers in this way would enable us to find particularly committed participants, in order to enable several design aspects that would require such commitment and that are difficult to achieve in large cohort studies. First, we needed a method that could recruit participants on a large scale, economically, and at a controlled rate so that blood samples could be processed by the laboratory on the day of receipt. The generational method appears to be able to do this. Although we ceased generational recruitment at 112 000 subjects, this was deliberate, based on resource availability, and there was no sign that we would ‘run out’ of eligible women. Probands in late generations were providing just as many next-generation recruits as had earlier generations. Thus, the recruitment was just short of self-sustaining over time if no new first-generation subjects had been added, and with a small addition of extra first-generation recruits from time to time could have been perpetuated almost indefinitely. The average cost per recruit (including laboratory costs, and including the costs for women approached who did not join the study) was £50 ($81).

Secondly, we wanted to obtain in the study questionnaire a great amount of detail on exposures in order to be able to analyse precise relationships and to investigate dose and duration response effects. Also, collection of exact numerical data rather than multiple-choice ranges gave maximum flexibility for future pooled analyses with other cohorts, if they have only collected grouped data. The questionnaire included more than 900 fields on 44 pages, and was filled in with a high degree of completeness (Morris *et al*, 2010). Similarly, we wished to collect blood specimens, both to obtain sufficient quantities of good-quality DNA to enable genetic and epigenetic analyses over the coming decades, and also to obtain plasma, particularly for hormone analyses, as sex hormones and probably growth-related hormones are critical to breast cancer aetiology (Hankinson *et al*, 2004). Blood samples were collected for >92% of the subjects, again reflecting their high degree of commitment.

Although a few large cohort studies have managed to collect updated questionnaire information every year or two on exposures in their cohort (e.g., Bernstein *et al*, 2002; Hays *et al*, 2003; Nurses’ Health Study, 2011), many have not, and the few collections of long-term repeat blood samples have been of a very limited scale (Nurses’ Health Study, 2011; Women's Health Initiative, 2011). Such information and samples are likely to be critical, however, to unravelling the aetiology of breast cancer and evaluating the effects of changing behaviours and environments, as the aetiological factors span most or all of a lifetime, from before menarche, perhaps prenatally, to after menopause (Hankinson *et al*, 2004). Obtaining repeat questionnaires and blood samples (and hence being able to assay hormone levels) from a high proportion of participants over decades is therefore important, but also likely to be extraordinarily difficult. So far, the generational recruitment method appears to have found cohort members sufficiently motivated to enable this. The study design includes, subject to the constraints of funding, repeated blood sampling periodically over the years.

It is also desirable to be able to obtain recorded information (e.g., diagnostic and therapeutic information from GP and hospital records) and images (e.g., mammograms) and to retrieve and use stored biological specimens (e.g., pathology specimens from tumours enabling histological and molecular classification of tumours). The information and written permission given by the study subjects have enabled all of these, with a good success rate in locating and obtaining the data and specimens (such acquisition is not yet complete, but currently is about 60–70%).

The choice of age group included in cohorts investigating the aetiology of breast cancer or many other cancers is almost inevitably a compromise between the scientific desire for prospectively gained exposure data and plasma samples from a young age, and the practical need for the study to accrue cancer outcomes, and hence results, sufficiently rapidly to be fundable. In practice, many cancer-oriented cohorts have recruited subjects from about age 50 and older, (van den Brandt *et al*, 1990; The Million Women Study Collaborative Group, 1999; Kolonel *et al*, 2000; Prorok *et al*, 2000; Schatzkin *et al*, 2001; Hays *et al*, 2003; White *et al*, 2004; Nurses’ Health Study, 2011), and uncommonly they have recruited down to age 25 or so (Bernstein *et al*, 2002; Nurses’ Health Study, 2011). We recruited at all ages from 16, in order to include both older subjects, who give more rapid results, and younger ones, who in the long term will enable more accurate, prospectively collected, exposure data and plasma samples through the reproductive years. Ideally, to encompass measurement of risk factors for breast cancer at all potentially relevant periods, a study would recruit prenatally and then follow up subjects throughout life, but we did not attempt this, and restricted the study to those age 16 or older at entry, because of the ethical difficulties in gaining consent to enrol children (or fetuses) for lifetime follow-up, the potential losses to adult follow-up if individuals had in practice been consented by their parents and not themselves, and the practical difficulties in obtaining and maintaining support and funding for a cancer study that would produce no direct cancer results for 30 or 40 years.

Focussing the design on investigation of breast cancer aetiology has the advantage of avoiding the compromises necessary in cohorts intended to investigate the causes of many diseases simultaneously (Elliott and Peakman, 2008), and probably adding to the commitment of the participants and hence to follow-up rates. It has also increased the extent of recruitment of high-risk individuals, because women with relatives with breast cancer have an incentive to join, and hence increased the power of the study, especially for genetic analyses (based on follow-up of the first 85 000 participants without prior breast cancer, we estimate that breast cancer risk in the cohort is about 1.6 times that in the general population). Thus, the proportion of our recruits aged 50–64 at entry whose mother (12%) or sister (5%) had had breast cancer was much greater than in relatively unselected women of the same ages undergoing breast screening in the United Kingdom (6 and 4%, respectively (The Million Women Study Collaborative Group, 1999)). It has the limitation, however, that although we are collecting data to enable investigation of other morbidity and mortality outcomes, the relevance of the exposure data available will diminish the more that the aetiology of the outcome is unlike that for breast cancer. Thus, for instance, the study data and samples are highly relevant to ovarian cancer or benign breast disease aetiology, but much less so to, say, diabetes. To maintain the focus on breast cancer, use of the samples is prioritised to this objective, and samples will only be used to investigate other outcomes if this does not divert them from breast cancer research.

The sources of the study subjects – Breakthrough Breast Cancer supporters, volunteers in response to publicity, and friends and family of existing members – are not a random sample of the general population. Those who are friends and family of the existing cohort members are also, on average, more similar to the probands than would be expected in members of the general population, which could be a defect if they were intended as controls in a case–control study, but is not a bias for a cohort design. Cohort studies do not need to represent the general population in order to be valid, and indeed many of the most valuable have been solely comprised of a particular group (e.g., doctors (Doll and Hill, 1954) or nurses (Nurses’ Health Study, 2011)). Nevertheless, narrow target groups can sometimes reduce the heterogeneity of exposures and hence power. The generational recruitment method proved surprisingly able to generate participants from a wide spectrum of society – the age and socioeconomic distribution, and to a lesser extent geographic distribution, of late-generation recruits to our cohort was wide-ranging and was about the same irrespective of the selective distribution of the proband generation.

The Generations study is currently in its early stages, and hence published outputs to date have been based on the data and blood samples gathered at recruitment rather than at follow-up. Sufficient follow-up is now accumulating to enable cohort analyses in the near future, and we are keen on collaborative uses of the materials, both as part of pooling projects and as collaborations with particular investigators (e.g., Murray *et al*, 2011). We are constructing linked databases that hold all of the questionnaire and biological sample data, including those generated by collaborators outside our Institute, to maximise the availability and scope of data available for future analyses. For laboratory analyses and other variables that require intensive efforts to obtain data (e.g., mammographic density), the assays and data acquisition are on a nested case–control basis. An Oversight Committee is in place to vet and decide upon the uses of the biological samples. We estimate that the power of the study is such that, for example, by 2020 for an exposure with 20% prevalence, the study will have 96% power to detect at *P*<0.05 a relative risk of 1.2 for breast cancer incidence, 85% power to detect a relative risk of 1.3 for oestrogen receptor-negative breast cancer and 78% power to detect a relative risk of 1.4 for triple-negative breast cancer.

In summary, the ‘generational’ recruitment method has enabled recruitment of a cohort of >110 000 women of all adult ages, who have provided detailed questionnaires and blood samples, and a high response rate to follow-up thus far, and who appear to have the commitment to the study to enable long-term high-quality data and biological samples to continue to be collected over decades to come.

## Figures and Tables

**Table 1:** Descriptive characteristics of cohort participants and UK general population

	**Participants in cohort**		**UK population**
**Characteristic**	**Number**	**Percent**	**Percent^a^**
*Age at entry to study (years)*			
16–24	5286	4.7	14.0
25–34	17 579	15.7	15.6
35–44	23 839	21.3	18.5
45–54	26 865	24.0	15.7
55–64	27 920	24.9	14.4
65–102	10 560	9.4	21.8
			
*Socioeconomic status (ACORN score) at entry* b			
A (highest)	51 568	46.0	25.4
B	12 641	11.3	11.5
C	31 875	28.5	27.4
D	8899	7.9	13.8
E (lowest)	6424	5.7	21.2
Unclassified^c^	112	0.1	—
Outside ACORN coverage^d^	530	0.5	—
			
*Region of residence at entry*			
South of England	48 641	43.4	34.5
East of England	12 443	11.1	9.3
Midlands	17 873	15.9	15.9
North of England	20 711	18.5	23.8
Scotland	7290	6.5	8.5
Northern Ireland	653	0.6	2.8
Wales	3908	3.5	4.9
Isle of Man, Channel Islands	530	0.5	0.4
			
*Family members in study*			
Mother or daughter(s)	22 814	20.4	—
Sister(s)	15 157	13.5	—
Aunt(s)	3028	2.7	—
Grandmother(s)	341	0.3	—
			
Total	112 049	100.0	100.0

a Including Isle of Man and Channel Islands for age and region of residence, but excluding those locations for ACORN score. Data from various years after 2000, depending on data availability. Age data are for females; the other data are for both sexes combined. ACORN data are for ages 16–74 from CACI (2009); the other data are for ages ⩾16 calculated from various government statistical sources.

b Socioeconomic score based on postcode of residence (CACI, 2009).

c Primarily communal residences such as student halls, and newly built (post-Census) properties.

d Resident in Isle of Man and Channel Islands, for which ACORN coding is not applicable.

**Table 2:** Recruitment ratios (number of new participants recruited directly by proband), by characteristics of proband

**Characteristic of proband**	**Number of probands**	**Number of recruits^a^**	**Recruitment ratio**
*Age at entry to study*			
16–24	3941	1808	0.46
25–34	13 390	9460	0.71
35–44	19 961	14 679	0.74
45–54	23 721	20 984	0.88
55–64	25 388	23 976	0.94
65–74	8501	6427	0.76
75–102	1286	771	0.60
			
*Socio-economic status (ACORN score) at entry* b			
A (highest)	45 133	40 984	0.91
B	10 851	8533	0.79
C	27 088	20 299	0.75
D	7314	4944	0.68
E (lowest)	5257	2882	0.55
Unclassified^c^	88	56	0.71
Outside ACORN coverage^d^	457	407	0.90
			
*Region at entry*			
South	42775	34935	0.82
East of England	10 333	8489	0.82
Midlands	15 339	12 894	0.84
North of England	17 425	13 675	0.78
Scotland	6055	4852	0.80
Northern Ireland	471	272	0.59
Wales	3333	2581	0.78
Isle of Man, Channel Islands	457	407	0.90
			
Total	96 188	78 105	0.81

a New participants recruited directly by proband.

b Socioeconomic score based on postcode of residence (CACI, 2009).

c Primarily communal residences such as student halls, and newly built (post-Census) properties.

d Resident in places (largely Isle of Man, Channel Islands) for which ACORN coding is not applicable.

**Table 3:** Age distribution of ⩾4th-generation recruits to the cohort for different age groups of first-generation probands

	**Distribution (%) of age (years) at recruitment in ⩾4th-generation recruits**						
						**Total**	
**Age (years) at recruitment of 1st** **generation^a^**	**16–34**	**35–44**	**45–54**	**55–64**	**65–102**	**%**	**Number**
1st generation aged 16–34 (*n*=4413)	18.1	20.0	24.5	28.1	9.3	100.0	5237
1st generation aged 35–44 (*n*=4427)	15.8	22.4	24.8	27.1	10.0	100.0	8038
1st generation aged 45–54 (*n*=4349)	16.2	19.8	26.8	26.3	10.9	100.0	16 284
1st generation aged 55–64 (*n*=4134)	16.4	19.4	24.7	28.8	10.7	100.0	17 456
1st generation aged 65–102 (*n*=1540)	14.2	17.6	25.3	29.7	13.2	100.0	4095
All ages 1st generation (*n*=18 863)	16.2	19.9	25.4	27.7	10.7	100.0	51 110

a i.e. the probands at the start of the recruitment chains.

**Table 4:** Socioeconomic distribution of ⩾4th-generation recruits to the cohort for different socioeconomic groups of first-generation probands

	**Distribution (%) of ACORN score in** ⩾**4th-generation recruits**						
						**Total**	
**ACORN^a^ score at recruitment of 1st generation^b^**	**A (highest)**	**B**	**C**	**D**	**E (lowest)**	**%**	**Number**
1st generation A (highest) (*n*=7471)	50.3	10.2	28.0	7.0	4.5	100.0	27 314
1st generation B (*n*=2752)	50.0	11.9	26.8	6.9	4.3	100.0	9063
1st generation C (*n*=5401)	48.2	10.5	28.8	7.1	5.4	100.0	10 373
1st generation D (*n*=1738)	49.1	10.2	28.0	8.4	4.3	100.0	2586
1st generation E (lowest) (*n*=1390)	45.1	13.8	27.4	7.3	6.4	100.0	951
All 1st generation (A–E) (*n*=18 752)	49.7	10.7	27.9	7.1	4.7	100.0	50 287

a Socioeconomic score based on postcode of residence (CACI, 2009).

b i.e. the probands at the start of the recruitment chains.
